# Revising traditional theory on the link between plant body size and fitness under competition: evidence from old-field vegetation

**DOI:** 10.1002/ece3.1001

**Published:** 2014-02-25

**Authors:** Amanda J Tracey, Lonnie W Aarssen

**Affiliations:** 1 Department of Biology, Queen's UniversityKingston, Ontario, K7L 3N6, Canada

**Keywords:** Coexistence, competition, fitness, local density, neighbor-removal, plant size, recruitment, trade-off

## Abstract

The selection consequences of competition in plants have been traditionally interpreted based on a “size-advantage” hypothesis – that is, under intense crowding/competition from neighbors, natural selection generally favors capacity for a relatively large plant body size. However, this conflicts with abundant data, showing that resident species body size distributions are usually strongly right-skewed at virtually all scales within vegetation. Using surveys within sample plots and a neighbor-removal experiment, we tested: (1) whether resident species that have a larger maximum potential body size (MAX) generally have more successful local individual recruitment, and thus greater local abundance/density (as predicted by the traditional size-advantage hypothesis); and (2) whether there is a general between-species trade-off relationship between MAX and capacity to produce offspring when body size is severely suppressed by crowding/competition – that is, whether resident species with a larger MAX generally also need to reach a larger minimum reproductive threshold size (MIN) before they can reproduce at all. The results showed that MIN had a positive relationship with MAX across resident species, and local density – as well as local density of just reproductive individuals – was generally greater for species with smaller MIN (and hence smaller MAX). In addition, the cleared neighborhoods of larger target species (which had relatively large MIN) generally had – in the following growing season – a lower ratio of conspecific recruitment within these neighborhoods relative to recruitment of other (i.e., smaller) species (which had generally smaller MIN). These data are consistent with an alternative hypothesis based on a ‘reproductive-economy-advantage’ – that is, superior fitness under competition in plants generally requires not larger potential body size, but rather superior capacity to recruit offspring that are in turn capable of producing grand-offspring – and hence transmitting genes to future generations – *despite* intense and persistent (cross-generational) crowding/competition from near neighbors. Selection for the latter is expected to favor relatively small minimum reproductive threshold size and hence – as a tradeoff – relatively small (not large) potential body size.

## Introduction

Competition is generally regarded as the most important organizing force affecting plant community structure and assembly (Tilman 1982). Light and soil resources are routinely limiting within natural vegetation, and resource acquisition is highly asymmetric and thus commonly dependent on relative plant size (Craine 2009). Many previous studies have confirmed that growth suppression is usually greater for relatively small species than for larger species when competing together in experimental mixtures (Gaudet and Keddy 1988; Keddy and Shipley 1989; Goldberg and Landa 1991; Keddy et al. 1994, 2002; Rosch et al. 1997; Keddy 2001). Traditional competition theory therefore is based on widespread support for a ‘size-advantage’ hypothesis – that is, intense competition between plants generally selects for a strategy that includes capacity to grow to a relatively large body size (Grime 1973; Grace 1990; Goldberg 1996). If this is true, then we can predict – for severely crowded vegetation near carrying capacity – that resident species with larger potential body sizes should generally transmit more gene copies to the next generation within the local community; that is, they should generally be more successful in recruiting offspring within the community and thus have greater numbers of resident plants, including mostly crowded juveniles, in addition to mature reproductive individuals.

A general positive relationship between life span and age at first reproduction is well known (Lacey 1986), but body size is one of the most phenotypically plastic traits in perennial species, and so older plants are not necessarily bigger. Variation in plant size, therefore, is commonly affected more by variation in the local intensity of crowding/competition from neighbors than by variation in age (Harper 1977). A recent review of relationships between body size and abundance suggests that most research in this area has been conducted on animals with little data available for plants, particularly for the relationship of interest here – the local size–density relationship (LSDR), where the abundance of each species is measured at the same location (White et al. 2007). In general, LSDRs show that body size explains little variation in abundance, possibly because LSDRs typically examine a relatively small range of body sizes (compared with global scales), thus accounting for relatively low *r*^2^ values (White et al. 2007). A few studies of forest vegetation, however, have shown significant negative between-species relationships for species body size versus local stem density (Condit 1988; Muller-Landau 2006), typically displaying as a reverse J-shape curve distribution. McCarthy and Bailey (1996), for example, reported this for all woody species in a mixed hardwood forest, where smaller size classes had up to 1000 stems, whereas larger size classes had only up to 10 individuals. Several other studies have confirmed this relationship, for example, in northern hardwood stands (Goodburn and Lorimer 1999) and in old growth spruce forests (Svensson and Jeglum 2001).

These and other recent studies raise doubts regarding the traditional view that evolution under selection from intense competition has generally favored a capacity for large plant body size. Chambers and Aarssen (2009) found that under the most crowded conditions within herbaceous angiosperm populations, the vast majority of the total offspring (seeds) available to represent the population in the next generation were produced by resident plants belonging to the second, third, and fourth smallest deciles of the plant size distribution. In addition, a recent multiple-species competition experiment with annuals (Neytcheva and Aarssen 2008) found that 97% of the between-species variation in reproductive (seed) output under intense competition was explained by variation in the number of survivors that, although suppressed weaklings, still managed to reproduce. Based on a recent survey of published literature – including for both short-lived semelparous and potentially longer-lived iteroparous species – Bonser (2013) reported that the efficiency of conversion of resources from vegetative tissue to reproductive output is generally higher (not lower) under more intense competition – contrary to traditional life history theory.

The above studies suggest that between-species variation in minimum reproductive threshold size (MIN) may be equally or even more important than between-species variation in maximum potential body size (MAX) in affecting between-species variation in capacity to transmit genes to future generations under intense crowding/competition. We explored this in this study of relationships between body size, reproduction, recruitment, and abundance for resident herbaceous species growing in a typical old-field meadow in southeastern Ontario, Canada. Specifically, we tested: (1) whether resident species that have a larger (MAX) generally have more successful local individual recruitment, and thus greater local abundance/density (as predicted by the traditional size-advantage hypothesis); and (2) whether there is a general between-species trade-off relationship between MAX and capacity to produce offspring when body size is severely suppressed by crowding/competition – that is, whether resident species with a larger MAX generally also need to reach a larger MIN before they can reproduce at all. Support for the latter would suggest that when crowding/competition from near neighbors is especially severe – with body sizes suppressed to the limit for the vast majority of resident plants – smaller species (contrary to traditional theory) may not have inferior fitness, as most (or virtually all) resident plants of larger species would be likely to fail in reaching their relatively large MIN sizes required to achieve any reproduction at all.

## Materials and Methods

### Study site

Field work was conducted from May to September in both 2010 and 2011, and from August to October in 2012 at the Queen's University Biological Station (QUBS), Chaffey's Locks, Ontario, Canada (44°33'N, 76°21'W). The study site is an old-field meadow approximately 2 ha in size, irregular in shape, and surrounded on all sides by mature woodland. The field was last tilled and sown about seventy years earlier with a hay mixture of timothy (*Phleum pratense* L.) and red clover (*Trifolium pratense* L.). The field had been mown for hay periodically up until summer 2008, and since then had been undisturbed by human activity, with the exception of occasional foot traffic and specimen collection by QUBS researchers. The plant community consists of perennial dicots, grasses, and sedges, all typical of old-field vegetation in the southeastern Ontario region. The most common species include *Phleum pratense* L., *Poa pratensis* L., *Vicia cracca* L., *Trifolium pratense* L., and *Ranunculus acris* L. A total of 43 resident vascular plant species have been recorded (Tracey and Aarssen 2011).

### Data collection – abundance

Between mid-June and mid-August 2010, abundance data were measured as counts of individuals (or ‘ramets’) for all vascular plant species present within 78 randomly positioned quadrats (1.0 × 1.0 m). For tufted, noncreeping sedges and grasses (e.g., *Phelum pratense* L.), both an individual tiller and a tuft of tillers were counted as an individual. For clonal rhizomatous species (e.g., *Poa pratensis* L.), just the local tiller or tuft of tillers that were visible aboveground were regarded as an individual. For stoloniferous species (*Fragaria virginiana* L, *Trifolium repens* L.), all connected tissues that could be seen aboveground were regarded as an individual. Within each 1 × 1 m quadrat, all individuals of each species were clipped at ground level and counted, with separate counts for reproductive and nonreproductive individuals. A reproductive individual was defined as one displaying flowers or indications of present or recent seed/fruit production. For each resident species, regardless of its MAX, most counted individuals were only a small fraction of MAX because of natural crowding/competition from near neighbors.

### Data collection – maximum potential body size

Plant species obviously differ in maximum potential body size that can be attained by a single rooted unit (which may be an individual genet or may be an individual ramet in a clonal species), but quantifying this accurately in situ for natural vegetation is complicated by uncontrolled sources of variation in genotype, age and local environmental conditions. Accordingly, MAX, like fitness, can only be recorded as a relative estimate. To estimate relative MAX for our study species, the field was surveyed in May 2011 using visual inspection to locate the five largest individuals (target plants) of each of the 35 species studied by Tracey and Aarssen (2011). Neighboring plants within a 50 cm radius around each target plant (defined as the target plant ‘neighborhood’) were clipped at ground level (with clippings left in place). Metal wire cages 1 m in diameter and 1 m high were constructed and attached to the ground, centered on each target plant, in order to prevent herbivory by deer and rabbits and to prevent aboveground growth of plants beyond the cages from extending into the target neighborhood area. In addition, the ground around the exterior perimeter of each cage was trenched with a spade to a depth of 25 cm in order to sever any roots of outside plants that might extend into the target neighborhood area. Dried hay was added to a depth of approximately five cm on the ground surrounding the target plant within each cage to minimize re-growth of cut vegetation and to minimize any increased moisture evaporation from the soil surface resulting from the reduced ground cover caused by removing neighboring vegetation. For species that were climbers (i.e., *Vicia cracca* L., *Cerastium arvense* L., etc.), target plants were able to climb up the side of the cage. The plots were visited weekly over the growing season to monitor the condition of target plants and to clip any emergent re-growth of vegetation within the target neighborhood.

Later in the growing season, each target species was harvested when plants reached the reproductive stage and had stopped growing in size, while showing visible signs of senescence (e.g., withering/brown tissue, fallen leaves). The plant was cut at ground level, and all aboveground biomass (including leaves that had fallen off) for each target plant was collected and dried in a drying oven for 7 days at 70°C. The plants were then weighed to obtain their dry mass, representing their MAX (for aboveground growth). Minimum reproductive threshold sizes (MIN) were recorded during 2009 as part of an earlier study (Tracey and Aarssen 2011) for the same 35 species growing as resident plants within the same community – and, again, where a reproductive individual was defined as one displaying flowers or indications of present or recent seed/fruit production.

### Data collection – recruitment

From August to October 2012 – 1 year after target plants were harvested – recruitment of individuals was recorded for the neighborhood areas within which vegetation had been removed around the target plants in the previous year. Recruitment was measured by cutting – at ground level – all vegetation growing within the 1-m diameter target neighborhoods and counting the number of individuals present for both the target species and other (nontarget) species.

### Data analyses

The between-species relationship for MAX versus MIN was analyzed with Type I linear regression, and reduced major axis (RMA) regression was used to test for departure of the slope from 1.0 (isometry). Linear regression was also used to examine between-species relationships for abundance versus MIN and versus MAX and to examine the relationship between the harvested biomass of target species (MAX) and the subsequent recruitment (in the following growing season) of target and nontarget species within the target plant neighborhoods. Both MIN and MAX, as well as the orthogonal residuals of their relationship, were used as multiple regression variables for predicting both total abundance and reproductive abundance. This analysis was based on automated model selection in R (dredge and step AIC functions for determining the top models with the best predictors (R Development Core Team 2008). All analyses were based on log-transformed data.

## Results

**Figure 1:**
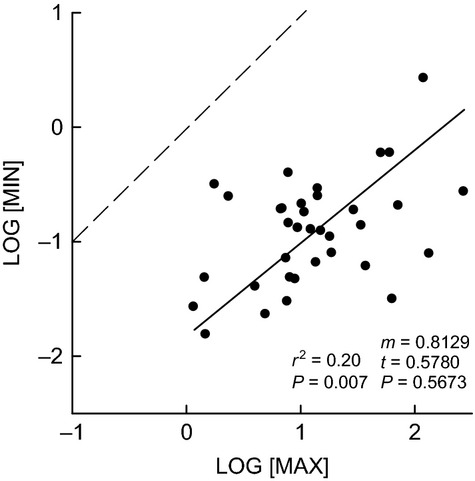
Between-species relationship for maximum versus minimum reproductive body size: Aboveground dry mass (g; log-transformed) of the largest individual growing without competition (MAX) recorded in ‘vegetation removal’ plots (selected from five treatment replicates per species) versus minimum reproductive threshold size (MIN; aboveground dry mass (g; log-transformed) of the smallest resident reproductive plant sampled within the community). *N* = 35; *r*^2^ and the associated *P*-values are from Type I linear regression analysis. Solid line is from RMA regression analysis; m = RMA slope; t- and associated *P*-value test for deviation from the null hypothesis of m = 1.0 (isometry). Dashed line is shown only for reference to the 1:1 line.

The study species varied in MAX by over two orders of magnitude, and MAX had a proportionately positive relationship with MIN, that is, with a slope not significantly different from 1.0 (Fig. 1). On average, across the 35 species – based on the raw data displayed in Fig. 1 – the smallest resident reproductive plants (MIN) within the community were suppressed by 98% on average (ranging from 82–99%) compared with species MAX recorded in the absence of near neighbors.

**Figure 2:**
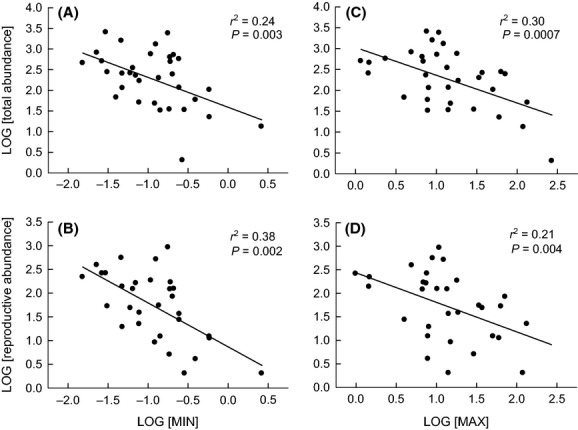
Between-species relationships for community abundance versus body size: Total community abundance (*n* = 33 species) and abundance for reproductive plants only (*n* = 31 species), based on ramet counts within 78–1.0 × 1.0 m sample plots, versus: (A) and (B) – minimum reproductive threshold size (MIN; aboveground dry mass (g) of the smallest resident reproductive plant sampled within the community); and (C) and (D) – maximum potential body size (MAX; aboveground dry mass (g) of the largest individual growing without competition recorded in ‘vegetation removal’ plots). All data are log-transformed. Note that (A) and (B) have the same *x*-axis, and (C) and (D) have the same *x*-axis. Similarly, (A) and (C) have the same *y*-axis, and (B) and (D) have the same *y*-axis. *r*^2^ and associated *P*-values are from Type I linear regression analyses. For (A) and (C), *n* = 33 as not all species collected for body size in 2009 were present within plots when recording abundance in 2010. For (B) and (D), *n* = 31 because some species had only nonreproductive plants present within surveyed plots in 2010.

More abundant species within sample plots – in terms of total numbers of resident individuals as well as counts for just reproductive individuals – were generally those with smaller MIN and smaller MAX (Fig. 2). A measure of the possible combined effects of MIN and MAX – based on the orthogonal residuals for their relationship in Fig. 1 – had no significant relationship with total abundance (linear regression of log-transformed data; *r*^2^ = 0.02, *P* = 0.828) or with reproductive abundance (linear regression of log-transformed data; *r*^2^ = 0.04, *P* = 0.257). In multiple regression analyses, a model containing only MIN was the best predictor of reproductive abundance (*r*^2^ = 0.356, *P* = 0.0003), and a model containing both MIN and MAX was the best predictor of total abundance (*r*^2^ = 0.361, *P* = 0.001; partial *r*_min_ = −0.17, *P* = 0.044; partial *r*_max_ = −0.19, *P* = 0.001). In the same analysis, a model with only MAX as a predictor of total abundance had a ΔAICc value ≤2 and as such should also be considered as a top model. Partial correlation analysis was also used, and the results agreed with the top models in multiple regression. The orthogonal residuals were removed from the analysis due to the high variance inflation factor (VIF) when used as a predictor variable.

**Figure 3:**
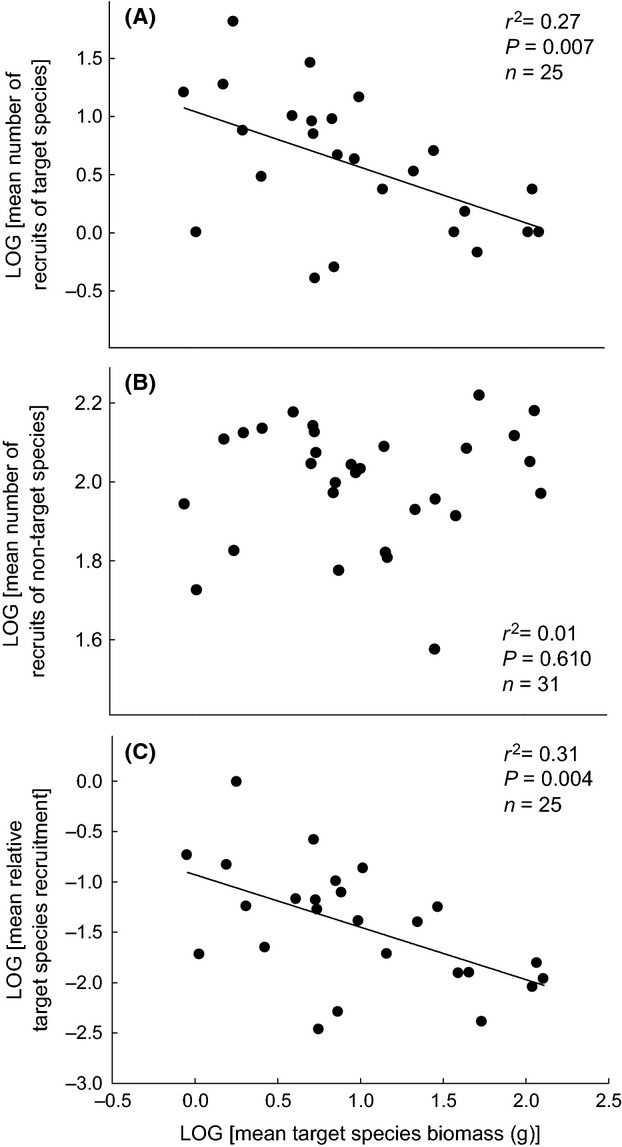
Recruitment success of resident species below cleared neighborhoods of target plants of different-sized resident species: Between-species relationships for mean (across-replicate) target species dry mass (g, aboveground) for individuals growing without competition from near neighbors (i.e., recorded in five replicate ‘vegetation removal’ plots) (*x*-axis) versus: (A) mean (across-replicate) number of recruits of target species within the target neighborhood 1 year after target harvest; (B) mean (across-replicate) number of recruits of nontarget species within the target neighborhood 1 year after target harvest; and (C) mean (across-replicate) relative target species recruitment, that is, (mean number of recruits of target species)/(mean number of recruits of nontarget species) within the target neighborhood 1 year after target harvest. Note that (A), (B), and (C) all have the same *x*-axis. All data are log-transformed. *r*^2^ and associated *P*-values are from Type I linear regression analyses. *N* values are less than 35 because target neighborhoods for some species set up in 2011 could not be re-located in 2012, and some target species had zero recruitment.

Mean conspecific recruitment of target species 1 year following removal of vegetation within their neighborhoods was generally greater for target species that had smaller mean target plant biomass (i.e., smaller MAX) (Fig. 3A). Mean recruitment of nontarget species within target neighborhoods, however, had no relationship with increasing mean target species biomass (Fig. 3B), and so target species with greater mean biomass generally had lower recruitment success within their own neighborhoods, relative to the recruitment of other (nontarget) species (Fig. 3C).

## Discussion

The structure of most natural vegetation contradicts the size-advantage hypothesis – that is, most species are relatively small, and size distributions of resident species within vegetation are right-skewed at virtually every scale (Aarssen and Schamp 2002; Niklas et al. 2003; Aarssen et al. 2006; Poorter et al. 2008; Moles et al. 2009; Schamp and Aarssen 2009; Dombroskie and Aarssen 2010; McGlone et al. 2010), including also in the present old-field study site (Tracey and Aarssen 2011). Even in later stages of succession, as larger species accumulate over time and cause the local competitive exclusion of some smaller species, other relatively small species take their place and species body size distributions typically remain strongly right-skewed (Schamp and Aarssen 2009; Waugh and Aarssen 2012). These findings have one or more of three profound implications: (1) recruitment success is not as limited by competition as is usually assumed for most natural vegetation; or (2) competition intensity between small and large species is weak because of niche differentiation mechanisms and/or opportunities for concurrent facilitation effects that are still poorly understood for plant communities; or (3) relatively small plant body size – contrary to traditional theory – does not impose inferior competitive fitness.

**Figure 4:**
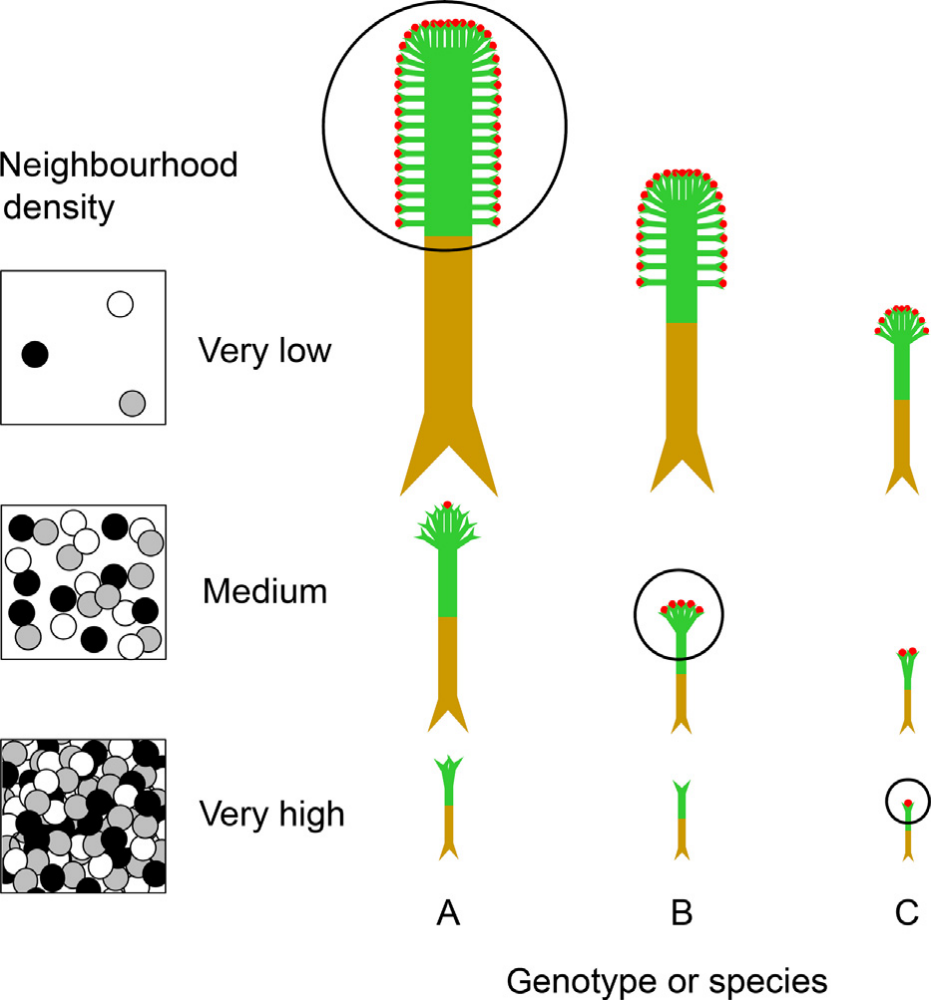
Symbolic representation of the ‘reproductive-economy-advantage’ hypothesis. Hypothetical plants (genotypes or species) A, B, and C are represented by differently colored circles (white/gray/black) within three square ‘plots’ showing different neighborhood densities of resident seeds, which is the only stage in which the three plants – as embryos – are all the same size. In each case, the ‘stick-plant’ symbols represent the relative body sizes of A, B, and C following emergence and growth to final developmental stage. The maximum potential body sizes (MAX) for A, B, and C can be expressed only when neighborhood density is very low (top row), where A has the largest MAX and hence the highest fecundity (indicated for each density by the collection of small red circles at the ends of plant ‘shoots’, delineated within the back circular outlines), and where it is thus favored by natural selection. Plant A therefore also has (as a trade-off) the largest minimum reproductive threshold size (MIN) (c.f. Fig. 1), which is expressed within a higher (intermediate)-density neighborhood (middle row). Here, plant B is favored by natural selection because its smaller MIN permits a higher fecundity than A, and its larger MAX permits a higher fecundity than C. Under very high neighbor density (bottom row), however, where all resident plants are severely suppressed in size, plant C has the highest fecundity (and is thus favored by natural selection) because it has the smallest MIN (which imposes, as a trade-off, the smallest MAX; c.f. Fig. 1). Under these conditions, plants of both A and B die without sex, because MIN for both is too large. Accordingly, contrary to the ‘size-advantage’ hypothesis, selection in favor of relatively large MAX (plant A) occurs, not under the most crowded conditions, but only within local neighborhoods where competition effects are relatively weak (top row) – because only here can MAX (and its potential fitness advantage) be realized. The preponderance of relatively small resident species within most natural vegetation, therefore, can be at least partially accounted for by a preponderance there of severely crowded neighborhoods (bottom row).

Actual measurement of the fitness of individuals in situ is of course not possible within natural vegetation. Accordingly, our data represent an estimate of differences in capacity of species with different body sizes to transmit genes to future generations. The latter – the focus of the present study – represents the concept of ‘reproductive economy’, that is, the capacity for at least some offspring production despite severe plant size suppression under competition (Aarssen 2008). Reproductive economy may be linked to a number of traits associated with relatively small species body size in plants, including selection for increased selfing rates, increased clonality, smaller seed size, earlier reproduction or smaller size at reproductive maturity (Aarssen et al. 2006; Aarssen 2008). According to the ‘reproductive-economy-advantage’ (REA) hypothesis, therefore success under competition in plants generally requires not larger potential body size, but rather superior capacity to recruit offspring that are in turn capable of producing grand-offspring – and hence transmitting genes to future generations – *despite* intense crowding/competition from near neighbors (Fig. 4).

The latter is reflected in MIN, and the present results show this to have a positive relationship with MAX across species (Fig. 1). In other words, species with a larger maximum potential body size generally also – as a trade-off – need to be proportionately larger before they can reproduce at all. This parallels the results of Tracey and Aarssen (2011) who found that species MIN was positively correlated with the maximum body size recorded for resident plants. The latter were growing in the presence of naturally occurring near neighbors, and so these body sizes were 28% smaller on average (across the 35 study species; data not shown) compared with the values for MAX (with near neighbors experimentally removed) recorded in the present study. Accordingly, the data analyzed here represent a closer approximation of the effects of between-species genetic variation on the relationship between minimum and maximum reproductive plant body sizes for these study species (Fig. 1).

To the best of our knowledge, these data – recorded for the resident species of a single plant community – are the first of their kind for any vegetation type. A previous neighbor-removal study in a similar nearby old-field community (Taylor and Aarssen 1990) showed that target plants (selected randomly) that had near neighbors left in place were 75–85% smaller in size (aboveground dry mass) at the end of the growing season compared with target plants that had near neighbors removed. The present data for MIN and MAX (Fig. 1), however, illustrate an unprecedented potential range of plasticity for reproductive plant size in old-field perennial species; remarkably, aboveground biomass values of the smallest reproductive plants for resident species were on average only 2% of their maximum potential aboveground biomass values.

For the same community of species as in the present study, Tracey and Aarssen (2011) reported a proportional relationship between MIN and maximum *resident* body size that is measured in the presence of natural neighbors. Our present results (Fig. 1) indicate that this isometry holds also for MIN versus maximum *potential* body size. Accordingly, both small and large species are capable of reproduction at the same fraction – 2% on average – of MAX. Note that MAX was 1.4 times greater on average across species compared with maximum resident body size reported by Tracey and Aarssen (2011), and these two size metrics also have an isometric relationship (*P* = 0.034; data not shown). In other words, by freeing the largest resident plants of each resident species from competition with near neighbors for one growing season (the present study), growth of aboveground biomass was released on average by the same proportionate amount regardless of species body size.

Our data also failed to support a central prediction associated with the size-advantage hypothesis. We found that larger resident species generally had lower (not higher) local density within the community (Fig. 2) – a novel finding for herbaceous vegetation. As well – probably because of the latter – larger resident species had less (not more) successful individual recruitment within local neighborhoods dominated by conspecific parent plants that were freed of near neighbors in the previous growing season (Fig. 3A). These data are instead consistent with the REA hypothesis suggested above (Fig. 4); that is, local density (within sample plots), as well as local density of just reproductive individuals, was generally greater for species with smaller MIN (Fig. 2A, B), and hence smaller MAX (Fig. 2C, D) as a trade-off (Fig. 1). [local density, reported here, however, was not related to species maximum *resident* body size – reported by Tracey and Aarssen 2011 (*P* = 0.13, all individuals; *P* = 0.19, reproductive individuals only; data not shown)]. In addition, the cleared neighborhoods of larger target species (which had relatively large MIN as a trade-off; Fig. 1) had a generally lower ratio of conspecific recruitment relative to recruitment of other (i.e., smaller) species (Fig. 3C) – because, we suggest, the latter had generally smaller MIN (Fig. 1). Tracey and Aarssen (2011) also reported, for the same community, that plot occupancy (number of plots in which a species was recorded as present) was negatively related to MIN, but this measure of abundance had no significant relationship with maximum resident body size (measured in the presence of natural neighbors).

In conclusion, our results add to a growing body of data calling into question the traditional size-advantage hypothesis for interpreting the selection consequences of competition in plants. Because of persistent crowding/competition within most natural herbaceous vegetation, the maximum potential body size for a resident plant can only rarely be achieved. And when it is – in the absence of extended survival through shade tolerance – this will usually be a consequence of locally weak (not intense) neighbor effects that were experienced fortuitously during the earlier seedling/juvenile establishment phases. According to the reproductive-economy-advantage hypothesis (Fig. 4), plant fitness under intense competition for herbaceous species is more commonly promoted therefore by the capacity of a plant to transmit genes to future generations *despite* severe offspring body size suppression – that is, with relatively small MIN, and hence (as a tradeoff) relatively small MAX. With greater reproductive economy therefore more offspring reproduce before death (Aarssen 2008), and although their individual sizes are severely suppressed (compared with the very few resident plants that manage to get closer to their MAX), their vast number means that they can collectively contribute far more to the production of offspring available for the next (grand-offspring) generation (Chambers and Aarssen 2009; Tracey and Aarssen 2011).

Future research is required to re-examine traditional views on how competition has influenced the evolution of plant species, and in particular, their body sizes. In fertile, relatively undisturbed, open habitats supporting species with relatively weak shade tolerance (as in the present study), relatively large potential plant body size should be favored by selection, we predict, only when local crowding/competition intensity from near neighbors is relatively weak – not severe, as traditional theory espouses. Only under these relatively rare circumstances will there be a size advantage, because only here will local resource availability per capita be sufficient to support the growth required to achieve a large body size, and hence high per capita reproductive output (Fig. 4). And, importantly, because the latter will generally also require (as a trade-off) a large minimum reproductive threshold size, offspring and grand-offspring will also need to enjoy relatively weak neighbor effects in order to reproduce before death – that is, grow large enough to be capable of producing future generations of offspring and subsequent descendants. Because such circumstances are so very rarely found in natural vegetation – that is, most resident plants die without sex because of severe crowding – this accounts, at least partially, we suggest, for why the vast majority of plant species, even in the most crowded habitats on earth, are relatively small.
